# Diaqua­bis(2-bromo­benzoato-κ*O*)bis­(*N*,*N*-diethyl­nicotinamide-κ*N* ^1^)nickel(II)

**DOI:** 10.1107/S1600536809021795

**Published:** 2009-06-13

**Authors:** Tuncer Hökelek, Filiz Yılmaz, Barış Tercan, F. Elif Özbek, Hacali Necefoğlu

**Affiliations:** a Department of Physics, Hacettepe University, 06800 Beytepe, Ankara, Turkey; b Department of Chemistry, Faculty of Science, Anadolu University, 26470 Yenibağlar, Eskişehir, Turkey; c Department of Physics, Karabük University, 78050 Karabük, Turkey; d Department of Chemistry, Kafkas University, 63100 Kars, Turkey

## Abstract

In the monomeric centrosymmetric title Ni^II^ complex, [Ni(C_7_H_4_BrO_2_)_2_(C_10_H_14_N_2_O)_2_(H_2_O)_2_], the Ni^II^ ion is located on an inversion center. The asymmetric unit contains one 2-bromo­benzoate ligand, one diethyl­nicotinamide (DENA) ligand and one coordinated water mol­ecule. The four O atoms in the equatorial plane around the Ni^II^ ion form a slightly distorted square-planar arrangement, while the slightly distorted octa­hedral coordination is completed by two N atoms of two DENA ligands in the axial positions. The dihedral angle between the benzene ring and the attached carboxyl­ate group is 87.73 (15)°, while the pyridine and benzene rings are oriented at a dihedral angle of 42.48 (7)°. In the crystal structure, O—H⋯O hydrogen bonds link the mol­ecules into a two-dimensional network parallel to (10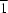). In addition, C—H⋯O hydrogen bonds are observed.

## Related literature

For general backgroud, see: Antolini *et al.* (1982 ▶); Bigoli *et al.* (1972 ▶); Nadzhafov *et al.* (1981 ▶); Shnulin *et al.* (1981 ▶). For related structures, see: Hökelek *et al.* (2009*a* ▶,*b* ▶,*c* ▶,*d* ▶); Özbek *et al.* (2009 ▶); Sertçelik *et al.* (2009*a* ▶,*b* ▶,*c* ▶); Tercan *et al.* (2009 ▶).
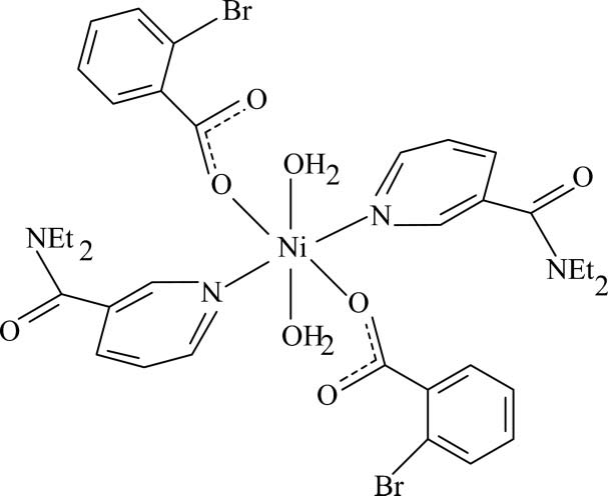


## Experimental

### 

#### Crystal data

[Ni(C_7_H_4_BrO_2_)_2_(C_10_H_14_N_2_O)_2_(H_2_O)_2_]
*M* *_r_* = 851.22
Monoclinic, 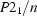

*a* = 12.8506 (3) Å
*b* = 10.3448 (2) Å
*c* = 14.9418 (4) Å
β = 114.004 (2)°
*V* = 1814.53 (8) Å^3^
*Z* = 2
Mo *K*α radiation
μ = 2.79 mm^−1^
*T* = 100 K
0.34 × 0.25 × 0.12 mm

#### Data collection

Bruker Kappa APEXII CCD area-detector diffractometer
Absorption correction: multi-scan (*SADABS*; Bruker, 2005 ▶) *T*_min_ = 0.442, *T*_max_ = 0.710
16814 measured reflections
4528 independent reflections
3603 reflections with *I* > 2σ(*I*)
*R*_int_ = 0.044

#### Refinement

*R*[*F* ^2^ > 2σ(*F* ^2^)] = 0.032
*wR*(*F* ^2^) = 0.078
*S* = 1.04
4528 reflections
233 parameters
H atoms treated by a mixture of independent and constrained refinement
Δρ_max_ = 0.78 e Å^−3^
Δρ_min_ = −0.78 e Å^−3^

### 

Data collection: *APEX2* (Bruker, 2007 ▶); cell refinement: *SAINT* (Bruker, 2007 ▶); data reduction: *SAINT*; program(s) used to solve structure: *SHELXS97* (Sheldrick, 2008 ▶); program(s) used to refine structure: *SHELXL97* (Sheldrick, 2008 ▶); molecular graphics: *ORTEP-3 for Windows* (Farrugia, 1997 ▶); software used to prepare material for publication: *WinGX* (Farrugia, 1999 ▶).

## Supplementary Material

Crystal structure: contains datablocks I, global. DOI: 10.1107/S1600536809021795/ci2824sup1.cif

Structure factors: contains datablocks I. DOI: 10.1107/S1600536809021795/ci2824Isup2.hkl

Additional supplementary materials:  crystallographic information; 3D view; checkCIF report

## Figures and Tables

**Table 1:** Selected bond lengths (Å)

Ni1—O1	2.0359 (14)
Ni1—O4	2.0818 (15)
Ni1—N1	2.1207 (17)

**Table 2:** Hydrogen-bond geometry (Å, °)

*D*—H⋯*A*	*D*—H	H⋯*A*	*D*⋯*A*	*D*—H⋯*A*
O4—H41⋯O3^i^	0.80 (3)	1.97 (3)	2.770 (2)	176 (4)
O4—H42⋯O2^ii^	0.78 (4)	1.88 (4)	2.623 (3)	161 (3)
C4—H4⋯O2^iii^	0.93	2.54	3.172 (3)	126
C8—H8⋯O3^i^	0.93	2.31	3.241 (3)	177
C10—H10⋯O1^iv^	0.93	2.47	3.392 (3)	172
C14—H14*A*⋯O2^v^	0.97	2.50	3.448 (3)	166
C14—H14*B*⋯O3^vi^	0.97	2.55	3.483 (3)	161

Symmetry codes: (i) 
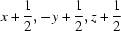
; (ii) 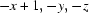; (iii) 
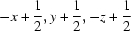
; (iv) 
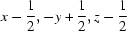
; (v) 
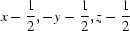
; (vi) 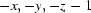.

**Fig. 1.**
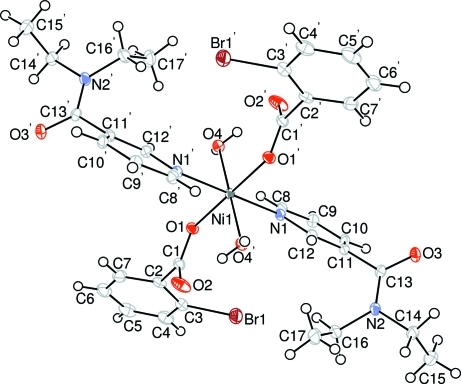
The molecular structure of the title compound, with the atom-numbering scheme. Displacement ellipsoids are drawn at the 50% probability level. Primed atoms are generated by the symmetry operator (1 - x, -y, -z).

## supplementary crystallographic information

### Comment

Transition metal complexes with biochemically active ligands frequently show interesting physical and/or chemical properties, as a result they may find applications in biological systems (Antolini *et al.*, 1982). The structural functions and coordination relationships of the arylcarboxylate ion in transition metal complexes of benzoic acid derivatives change depending on the nature and position of the substituent groups on the benzene ring, the nature of the additional ligand molecule or solvent, and the medium of the synthesis (Nadzhafov *et al.*, 1981; Shnulin *et al.*, 1981). The nicotinic acid derivative *N*,*N*-diethylnicotinamide (DENA) is an important respiratory stimulant (Bigoli *et al.*, 1972).

The structure determination of the title compound, (I), a nickel complex with two 2-bromobenzoate (BB), two diethylnicotinamide (DENA) ligands and two water molecules, was undertaken in order to determine the properties of the ligands and also to compare the results obtained with those reported previously.

Compound (I) is a monomeric complex, with the Ni^II^ ion on a centre of symmetry (Fig. 1). All ligands are monodentate. The four O atoms (O1, O4, and the symmetry-related atoms, O1', O4') in the equatorial plane around the Ni atom form a slightly distorted square-planar arrangement, while the slightly distorted octahedral coordination is completed by two N atoms of two DENA ligands (N1, N1') in axial positions (Table 1 and Fig. 1).

The near equality of C1—O1 [1.267 (3) Å] and C1—O2 [1.240 (3) Å] bonds in the carboxylate group indicates a delocalized bonding arrangement, rather than localized single and double bonds, and may be compared with the corresponding distances: 1.262 (3) and 1.249 (3) Å in [Mn(DENA)_2_(C_8_H_5_O_3_)_2_(H_2_O)_2_], (II) (Sertçelik *et al.*, 2009*a*), 1.263 (4) and 1.249 (4) Å in [Ni(DENA)_2_(C_8_H_5_O_3_)_2_(H_2_O)_2_], (III) (Sertçelik *et al.*, 2009*b*), 1.262 (5) and 1.257 (5) Å in [Co(DENA)_2_(C_8_H_5_O_3_)_2_(H_2_O)_2_], (IV) (Sertçelik *et al.*, 2009*c*), 1.244 (4) and 1.270 (4) Å in [Co(NA)_2_(H_2_O)_4_](C_7_H_4_FO_2_)_2_, (V) (Özbek *et al.*, 2009), 1.284 (2), 1.248 (2) and 1.278 (2), 1.241 (2) Å in [Zn(NA)_2_(C_8_H_8_NO_2_)_2_], (VI) (Tercan *et al.*, 2009), 1.267 (3) and 1.258 (3) Å in [Ni(NA)_2_(C_7_H_4_ClO_2_)_2_(H_2_O)_2_], (VII) (Hökelek *et al.*, 2009*a*), 1.263 (2) and 1.240 (2) Å in [Zn(DENA)_2_(C_7_H_4_BrO_2_)_2_(H_2_O)_2_], (VIII) (Hökelek *et al.*, 2009*b*), 1.2611 (17) and 1.2396 (19) Å in [Mn(DENA)_2_(C_7_H_4_BrO_2_)_2_(H_2_O)_2_], (IX) (Hökelek *et al.*, 2009*c*) and 1.2616 (17) and 1.2435 (18) Å in [Ni(DENA)_2_(C_7_H_4_ClO_2_)_2_(H_2_O)_2_], (*X*) (Hökelek *et al.*, 2009d). In (I), the average Ni—O bond length is 2.0589 (15) Å and the Ni1 atom is displaced out of the least-squares plane of the carboxylate group (O1/C1/O2) by 0.291 (1) Å. The dihedral angle between the planar carboxylate group and the benzene ring A (C2-C7) is 87.73 (15)°, while that between rings A and B (N1/C8-C12) is 42.48 (7)°.

In the crystal structure, intermolecular O—H···O hydrogen bonds (Table 2) link the molecules into a two-dimensional network parallel to the (1 0 1). In addition, C—H···O hydrogen bonds are observed.

### Experimental

The title compound was prepared by the reaction of NiSO_4_.6H_2_O (1.31 g, 5 mmol) in H_2_O (20 ml) and i>N,N-diethylnicotinamide (1.78 g, 10 mmol) in H_2_O (20 ml) with sodium 2-bromobenzoate (2.23 g, 10 mmol) in H_2_O (50 ml). The mixture was filtered and set aside to crystallize at ambient temperature for 2 d, giving blue single crystals.

### Refinement

H atoms of water molecule were located in difference Fourier maps and refined isotropically. The remaining H atoms were positioned geometrically with C-H = 0.93, 0.97 and 0.96 Å for aromatic, methylene and methyl H atoms and constrained to ride on their parent atoms, with *U*_iso_(H) = *xU*_eq_(C), where *x* = 1.5 for methyl H and *x* = 1.2 for all other H atoms.

### Crystal data

**Table:**

[Ni(C_7_H_4_BrO_2_)_2_(C_10_H_14_N_2_O)_2_(H_2_O)_2_]	*F*(000) = 868
*M**_r_* = 851.22	*D*_x_ = 1.558 Mg m^−^^3^
Monoclinic, *P*2_1_/*n*	Mo *K*α radiation, λ = 0.71073 Å
Hall symbol: -P 2yn	Cell parameters from 5301 reflections
*a* = 12.8506 (3) Å	θ = 2.5–28.3°
*b* = 10.3448 (2) Å	µ = 2.79 mm^−^^1^
*c* = 14.9418 (4) Å	*T* = 100 K
β = 114.004 (2)°	Block, blue
*V* = 1814.53 (8) Å^3^	0.34 × 0.25 × 0.12 mm
*Z* = 2	

### Data collection

**Table:**

Bruker Kappa APEXII CCD area-detector diffractometer	4528 independent reflections
Radiation source: fine-focus sealed tube	3603 reflections with *I* > 2σ(*I*)
graphite	*R*_int_ = 0.044
φ and ω scans	θ_max_ = 28.4°, θ_min_ = 1.8°
Absorption correction: multi-scan (*SADABS*; Bruker, 2005)	*h* = −17→17
*T*_min_ = 0.442, *T*_max_ = 0.710	*k* = −13→13
16814 measured reflections	*l* = −19→18

### Refinement

**Table:**

Refinement on *F*^2^	Primary atom site location: structure-invariant direct methods
Least-squares matrix: full	Secondary atom site location: difference Fourier map
*R*[*F*^2^ > 2σ(*F*^2^)] = 0.032	Hydrogen site location: inferred from neighbouring sites
*wR*(*F*^2^) = 0.078	H atoms treated by a mixture of independent and constrained refinement
*S* = 1.04	*w* = 1/[σ^2^(*F*_o_^2^) + (0.0334*P*)^2^ + 0.7499*P*] where *P* = (*F*_o_^2^ + 2*F*_c_^2^)/3
4528 reflections	(Δ/σ)_max_ = 0.001
233 parameters	Δρ_max_ = 0.78 e Å^−^^3^
0 restraints	Δρ_min_ = −0.77 e Å^−^^3^

### Special details

**Table:**

Geometry. All e.s.d.'s (except the e.s.d. in the dihedral angle between two l.s. planes) are estimated using the full covariance matrix. The cell e.s.d.'s are taken into account individually in the estimation of e.s.d.'s in distances, angles and torsion angles; correlations between e.s.d.'s in cell parameters are only used when they are defined by crystal symmetry. An approximate (isotropic) treatment of cell e.s.d.'s is used for estimating e.s.d.'s involving l.s. planes.
Refinement. Refinement of *F*^2^ against ALL reflections. The weighted *R*-factor *wR* and goodness of fit *S* are based on *F*^2^, conventional *R*-factors *R* are based on *F*, with *F* set to zero for negative *F*^2^. The threshold expression of *F*^2^ > σ(*F*^2^) is used only for calculating *R*-factors(gt) *etc*. and is not relevant to the choice of reflections for refinement. *R*-factors based on *F*^2^ are statistically about twice as large as those based on *F*, and *R*- factors based on ALL data will be even larger.

### Fractional atomic coordinates and isotropic or equivalent isotropic displacement parameters (Å^2^)

**Table:**

	*x*	*y*	*z*	*U*_iso_*/*U*_eq_	
Br1	0.17947 (2)	0.14287 (2)	0.072440 (17)	0.02619 (8)	
Ni1	0.5000	0.0000	0.0000	0.01120 (9)	
O1	0.46945 (12)	0.05353 (14)	0.11821 (10)	0.0154 (3)	
O2	0.37193 (16)	−0.11792 (15)	0.13480 (13)	0.0270 (4)	
O3	0.07077 (13)	0.11718 (14)	−0.39530 (10)	0.0173 (3)	
O4	0.61679 (13)	0.15121 (15)	0.03504 (12)	0.0146 (3)	
H41	0.607 (3)	0.219 (3)	0.056 (2)	0.036 (9)*	
H42	0.621 (3)	0.159 (3)	−0.015 (3)	0.040 (9)*	
N1	0.36374 (15)	0.12018 (16)	−0.08963 (12)	0.0134 (4)	
N2	0.01992 (15)	−0.00539 (17)	−0.29453 (12)	0.0167 (4)	
C1	0.41035 (18)	−0.0066 (2)	0.15502 (15)	0.0156 (4)	
C2	0.38690 (18)	0.0695 (2)	0.23103 (15)	0.0163 (4)	
C3	0.29000 (19)	0.1455 (2)	0.20453 (16)	0.0196 (4)	
C4	0.2709 (2)	0.2248 (2)	0.27162 (17)	0.0247 (5)	
H4	0.2060	0.2761	0.2521	0.030*	
C5	0.3501 (2)	0.2258 (2)	0.36754 (18)	0.0278 (5)	
H5	0.3394	0.2799	0.4128	0.033*	
C6	0.4451 (2)	0.1473 (2)	0.39693 (18)	0.0269 (5)	
H6	0.4967	0.1462	0.4622	0.032*	
C7	0.4636 (2)	0.0699 (2)	0.32851 (16)	0.0217 (5)	
H7	0.5281	0.0178	0.3484	0.026*	
C8	0.36086 (18)	0.2475 (2)	−0.07399 (15)	0.0158 (4)	
H8	0.4205	0.2842	−0.0210	0.019*	
C9	0.27291 (18)	0.3269 (2)	−0.13331 (16)	0.0173 (4)	
H9	0.2747	0.4151	−0.1207	0.021*	
C10	0.18249 (17)	0.2735 (2)	−0.21145 (15)	0.0161 (4)	
H10	0.1233	0.3249	−0.2531	0.019*	
C11	0.18272 (17)	0.1406 (2)	−0.22590 (14)	0.0142 (4)	
C12	0.27482 (17)	0.0686 (2)	−0.16478 (14)	0.0142 (4)	
H12	0.2753	−0.0197	−0.1762	0.017*	
C13	0.08624 (17)	0.08196 (19)	−0.31130 (15)	0.0139 (4)	
C14	−0.07886 (18)	−0.0564 (2)	−0.37834 (16)	0.0187 (4)	
H14A	−0.0945	−0.1439	−0.3640	0.022*	
H14B	−0.0611	−0.0595	−0.4355	0.022*	
C15	−0.18402 (19)	0.0266 (2)	−0.40082 (18)	0.0251 (5)	
H15A	−0.2463	−0.0081	−0.4566	0.038*	
H15B	−0.1687	0.1133	−0.4148	0.038*	
H15C	−0.2036	0.0270	−0.3453	0.038*	
C16	0.0277 (2)	−0.0429 (2)	−0.19692 (16)	0.0259 (5)	
H16A	−0.0472	−0.0364	−0.1961	0.031*	
H16B	0.0776	0.0171	−0.1486	0.031*	
C17	0.0725 (2)	−0.1794 (3)	−0.16850 (19)	0.0348 (6)	
H17A	0.0684	−0.2022	−0.1077	0.052*	
H17B	0.1502	−0.1836	−0.1611	0.052*	
H17C	0.0271	−0.2385	−0.2187	0.052*	

### Atomic displacement parameters (Å^2^)

**Table:**

	*U*^11^	*U*^22^	*U*^33^	*U*^12^	*U*^13^	*U*^23^
Br1	0.02396 (13)	0.03283 (15)	0.02130 (13)	0.00577 (10)	0.00871 (9)	0.00345 (10)
Ni1	0.01157 (17)	0.01200 (18)	0.00955 (17)	−0.00007 (13)	0.00379 (13)	−0.00031 (13)
O1	0.0171 (7)	0.0169 (7)	0.0139 (7)	−0.0024 (6)	0.0080 (6)	−0.0022 (6)
O2	0.0446 (11)	0.0170 (8)	0.0312 (9)	−0.0089 (7)	0.0275 (8)	−0.0057 (7)
O3	0.0209 (8)	0.0158 (7)	0.0119 (7)	−0.0003 (6)	0.0032 (6)	0.0022 (6)
O4	0.0173 (7)	0.0128 (8)	0.0139 (8)	−0.0008 (6)	0.0065 (6)	−0.0006 (6)
N1	0.0146 (8)	0.0135 (8)	0.0118 (8)	0.0001 (7)	0.0052 (7)	0.0011 (6)
N2	0.0174 (9)	0.0198 (9)	0.0112 (8)	−0.0032 (7)	0.0041 (7)	−0.0005 (7)
C1	0.0171 (10)	0.0166 (10)	0.0142 (10)	0.0028 (8)	0.0077 (8)	0.0016 (8)
C2	0.0209 (10)	0.0153 (11)	0.0184 (10)	−0.0030 (8)	0.0137 (9)	−0.0019 (8)
C3	0.0225 (11)	0.0203 (11)	0.0185 (11)	−0.0006 (9)	0.0110 (9)	0.0011 (9)
C4	0.0306 (13)	0.0230 (12)	0.0286 (13)	0.0011 (10)	0.0203 (11)	−0.0026 (10)
C5	0.0391 (14)	0.0261 (13)	0.0279 (13)	−0.0078 (11)	0.0237 (11)	−0.0110 (10)
C6	0.0300 (13)	0.0343 (14)	0.0189 (11)	−0.0101 (11)	0.0126 (10)	−0.0063 (10)
C7	0.0194 (11)	0.0260 (12)	0.0215 (11)	−0.0024 (9)	0.0101 (9)	−0.0025 (9)
C8	0.0172 (10)	0.0155 (10)	0.0142 (10)	−0.0013 (8)	0.0058 (8)	−0.0013 (8)
C9	0.0199 (10)	0.0128 (10)	0.0180 (10)	−0.0001 (8)	0.0064 (8)	−0.0002 (8)
C10	0.0159 (10)	0.0175 (10)	0.0135 (10)	0.0029 (8)	0.0046 (8)	0.0027 (8)
C11	0.0153 (10)	0.0159 (10)	0.0111 (9)	−0.0013 (8)	0.0052 (8)	0.0006 (8)
C12	0.0154 (10)	0.0146 (10)	0.0127 (10)	−0.0003 (8)	0.0058 (8)	0.0002 (8)
C13	0.0137 (9)	0.0109 (9)	0.0144 (10)	0.0021 (8)	0.0029 (8)	0.0009 (8)
C14	0.0189 (10)	0.0185 (11)	0.0161 (10)	−0.0049 (9)	0.0043 (8)	−0.0026 (8)
C15	0.0193 (11)	0.0290 (13)	0.0235 (12)	0.0002 (9)	0.0052 (9)	0.0036 (10)
C16	0.0253 (12)	0.0369 (14)	0.0144 (10)	−0.0126 (10)	0.0069 (9)	0.0000 (10)
C17	0.0284 (13)	0.0422 (16)	0.0254 (13)	−0.0081 (12)	0.0022 (11)	0.0177 (11)

### Geometric parameters (Å, °)

**Table:**

Br1—C3	1.906 (2)	C7—H7	0.93
Ni1—O1	2.0359 (14)	C8—N1	1.341 (3)
Ni1—O1^i^	2.0359 (14)	C8—H8	0.93
Ni1—O4	2.0818 (15)	C9—C8	1.387 (3)
Ni1—O4^i^	2.0818 (15)	C9—H9	0.93
Ni1—N1	2.1207 (17)	C10—C9	1.384 (3)
Ni1—N1^i^	2.1207 (17)	C10—H10	0.93
O1—C1	1.267 (3)	C11—C10	1.392 (3)
O2—C1	1.240 (3)	C11—C12	1.382 (3)
O3—C13	1.243 (2)	C11—C13	1.499 (3)
O4—H41	0.80 (3)	C12—N1	1.344 (3)
O4—H42	0.78 (3)	C12—H12	0.93
N2—C13	1.334 (3)	C14—C15	1.519 (3)
N2—C14	1.471 (3)	C14—H14A	0.97
N2—C16	1.473 (3)	C14—H14B	0.97
C2—C1	1.510 (3)	C15—H15A	0.96
C2—C7	1.387 (3)	C15—H15B	0.96
C3—C2	1.387 (3)	C15—H15C	0.96
C3—C4	1.392 (3)	C16—C17	1.518 (4)
C4—C5	1.379 (3)	C16—H16A	0.97
C4—H4	0.93	C16—H16B	0.97
C5—H5	0.93	C17—H17A	0.96
C6—C5	1.381 (4)	C17—H17B	0.96
C6—C7	1.393 (3)	C17—H17C	0.96
C6—H6	0.93		
			
O1—Ni1—O1^i^	180.00 (7)	C2—C7—H7	119.6
O1—Ni1—O4	87.20 (6)	C6—C7—H7	119.6
O1^i^—Ni1—O4	92.80 (6)	N1—C8—C9	122.93 (19)
O1—Ni1—O4^i^	92.80 (6)	N1—C8—H8	118.5
O1^i^—Ni1—O4^i^	87.20 (6)	C9—C8—H8	118.5
O1—Ni1—N1	89.27 (6)	C8—C9—H9	120.4
O1^i^—Ni1—N1	90.73 (6)	C10—C9—C8	119.3 (2)
O1—Ni1—N1^i^	90.73 (6)	C10—C9—H9	120.4
O1^i^—Ni1—N1^i^	89.27 (6)	C9—C10—C11	118.11 (19)
O4^i^—Ni1—O4	180.00 (9)	C9—C10—H10	120.9
O4—Ni1—N1	92.52 (6)	C11—C10—H10	120.9
O4^i^—Ni1—N1	87.48 (6)	C10—C11—C13	118.60 (18)
O4—Ni1—N1^i^	87.48 (6)	C12—C11—C10	119.01 (19)
O4^i^—Ni1—N1^i^	92.52 (6)	C12—C11—C13	122.28 (18)
N1^i^—Ni1—N1	180.00 (16)	N1—C12—C11	123.15 (19)
C1—O1—Ni1	127.15 (13)	N1—C12—H12	118.4
Ni1—O4—H41	123 (2)	C11—C12—H12	118.4
Ni1—O4—H42	99 (2)	O3—C13—N2	122.44 (18)
H41—O4—H42	112 (3)	O3—C13—C11	118.57 (18)
C8—N1—Ni1	122.69 (14)	N2—C13—C11	118.99 (18)
C8—N1—C12	117.44 (18)	N2—C14—C15	111.57 (18)
C12—N1—Ni1	119.87 (14)	N2—C14—H14A	109.3
C13—N2—C14	118.61 (17)	N2—C14—H14B	109.3
C13—N2—C16	124.98 (18)	C15—C14—H14A	109.3
C14—N2—C16	115.85 (18)	C15—C14—H14B	109.3
O1—C1—C2	114.11 (18)	H14A—C14—H14B	108.0
O2—C1—O1	126.8 (2)	C14—C15—H15A	109.5
O2—C1—C2	119.06 (19)	C14—C15—H15B	109.5
C3—C2—C1	120.77 (19)	C14—C15—H15C	109.5
C7—C2—C1	121.2 (2)	H15A—C15—H15B	109.5
C7—C2—C3	118.0 (2)	H15A—C15—H15C	109.5
C2—C3—Br1	119.46 (17)	H15B—C15—H15C	109.5
C2—C3—C4	121.9 (2)	N2—C16—C17	112.8 (2)
C4—C3—Br1	118.60 (18)	N2—C16—H16A	109.0
C3—C4—H4	120.6	N2—C16—H16B	109.0
C5—C4—C3	118.8 (2)	C17—C16—H16A	109.0
C5—C4—H4	120.6	C17—C16—H16B	109.0
C5—C6—C7	119.8 (2)	H16A—C16—H16B	107.8
C5—C6—H6	120.1	C16—C17—H17A	109.5
C7—C6—H6	120.1	C16—C17—H17B	109.5
C4—C5—C6	120.6 (2)	C16—C17—H17C	109.5
C4—C5—H5	119.7	H17A—C17—H17B	109.5
C6—C5—H5	119.7	H17A—C17—H17C	109.5
C2—C7—C6	120.8 (2)	H17B—C17—H17C	109.5
			
O4^i^—Ni1—O1—C1	8.54 (17)	C1—C2—C7—C6	175.5 (2)
O4—Ni1—O1—C1	−171.46 (17)	C3—C2—C7—C6	−1.8 (3)
N1^i^—Ni1—O1—C1	−84.02 (17)	Br1—C3—C2—C1	5.0 (3)
N1—Ni1—O1—C1	95.98 (17)	Br1—C3—C2—C7	−177.63 (16)
O1—Ni1—N1—C8	58.46 (17)	C4—C3—C2—C1	−174.6 (2)
O1^i^—Ni1—N1—C8	−121.54 (17)	C4—C3—C2—C7	2.7 (3)
O1—Ni1—N1—C12	−120.53 (16)	Br1—C3—C4—C5	179.28 (18)
O1^i^—Ni1—N1—C12	59.47 (16)	C2—C3—C4—C5	−1.1 (4)
O4^i^—Ni1—N1—C8	151.29 (17)	C3—C4—C5—C6	−1.5 (4)
O4—Ni1—N1—C8	−28.71 (17)	C5—C6—C7—C2	−0.7 (4)
O4^i^—Ni1—N1—C12	−27.70 (16)	C7—C6—C5—C4	2.4 (4)
O4—Ni1—N1—C12	152.30 (16)	C9—C8—N1—C12	−2.1 (3)
Ni1—O1—C1—O2	10.3 (3)	C9—C8—N1—Ni1	178.91 (16)
Ni1—O1—C1—C2	−169.71 (13)	C10—C9—C8—N1	1.1 (3)
C14—N2—C13—O3	−3.5 (3)	C11—C10—C9—C8	1.4 (3)
C14—N2—C13—C11	176.21 (18)	C12—C11—C10—C9	−2.8 (3)
C16—N2—C13—O3	−174.5 (2)	C13—C11—C10—C9	−179.0 (2)
C16—N2—C13—C11	5.1 (3)	C10—C11—C12—N1	1.9 (3)
C13—N2—C14—C15	−88.5 (2)	C13—C11—C12—N1	177.95 (19)
C16—N2—C14—C15	83.4 (2)	C10—C11—C13—O3	61.2 (3)
C13—N2—C16—C17	−110.3 (2)	C10—C11—C13—N2	−118.5 (2)
C14—N2—C16—C17	78.4 (2)	C12—C11—C13—O3	−114.9 (2)
C3—C2—C1—O2	−88.9 (3)	C12—C11—C13—N2	65.5 (3)
C7—C2—C1—O2	93.8 (3)	C11—C12—N1—C8	0.5 (3)
C3—C2—C1—O1	91.1 (2)	C11—C12—N1—Ni1	179.58 (16)
C7—C2—C1—O1	−86.2 (2)		

Symmetry codes: (i) −*x*+1, −*y*, −*z*.

### Hydrogen-bond geometry (Å, °)

**Table:**

*D*—H···*A*	*D*—H	H···*A*	*D*···*A*	*D*—H···*A*
O4—H41···O3^ii^	0.80 (3)	1.97 (3)	2.770 (2)	176 (4)
O4—H42···O2^i^	0.78 (4)	1.88 (4)	2.623 (3)	161 (3)
C4—H4···O2^iii^	0.93	2.54	3.172 (3)	126
C8—H8···O3^ii^	0.93	2.31	3.241 (3)	177
C10—H10···O1^iv^	0.93	2.47	3.392 (3)	172
C14—H14A···O2^v^	0.97	2.50	3.448 (3)	166
C14—H14B···O3^vi^	0.97	2.55	3.483 (3)	161

Symmetry codes: (ii) *x*+1/2, −*y*+1/2, *z*+1/2; (i) −*x*+1, −*y*, −*z*; (iii) −*x*+1/2, *y*+1/2, −*z*+1/2; (iv) *x*−1/2, −*y*+1/2, *z*−1/2; (v) *x*−1/2, −*y*−1/2, *z*−1/2; (vi) −*x*, −*y*, −*z*−1.
